# Exploring Factors Influencing Changes in Incidence and Severity of Multisystem Inflammatory Syndrome in Children

**DOI:** 10.3390/pathogens12080997

**Published:** 2023-07-30

**Authors:** Pasquale Castaldo, Gabriele d’Alanno, Giovanni Battista Biserni, Mattia Moratti, Francesca Conti, Marianna Fabi, Marcello Lanari

**Affiliations:** 1 Specialty School of Pediatrics, Alma Mater Studiorum, University of Bologna, 40138 Bologna, Italy; pasquale.castaldo2@studio.unibo.it (P.C.); gabriele.dalanno@studio.unibo.it (G.d.); mattia.moratti@studio.unibo.it (M.M.); 2 UOC Territorial Pediatric Unit, AUSL Bologna, 40124 Bologna, Italy; 3 Pediatric Unit, IRCCS Azienda Ospedaliero-Universitaria di Bologna, 40138 Bologna, Italy; francesca.conti27@unibo.it; 4 Pediatric Emergency Unit, IRCCS Azienda Ospedaliero-Universitaria di Bologna, 40138 Bologna, Italy; marianna.fabi@aosp.bo.it (M.F.); marcello.lanari@unibo.it (M.L.)

**Keywords:** MIS-C, SARS-CoV-2, Omicron, VoCs

## Abstract

Multisystem inflammatory syndrome (MIS-C) is a rare condition associated with COVID-19 affecting children, characterized by severe and aberrant systemic inflammation leading to nonspecific symptoms, such as gastrointestinal, cardiac, respiratory, hematological, and neurological disorders. In the last year, we have experienced a progressive reduction in the incidence and severity of MIS-C, reflecting the worldwide trend. Thus, starting from the overall trend in the disease in different continents, we reviewed the literature, hypothesizing the potential influencing factors contributing to the reduction in cases and the severity of MIS-C, particularly the vaccination campaign, the spread of different SARS-CoV-2 variants (VOCs), and the changes in human immunological response. The decrease in the severity of MIS-C and its incidence seem to be related to a combination of different factors rather than a single cause. Maturation of an immunological memory to SARS-CoV-2 over time, the implication of mutations of key amino acids of S protein in VOCs, and the overall immune response elicited by vaccination over the loss of neutralization of vaccines to VOCs seem to play an important role in this change.

## 1. Introduction

Since late 2019, SARS-CoV-2 infection has rapidly spread across the globe. Shortly after the first wave (March–April 2020), cases of a new nosographic entity, characterized by SARS-CoV-2-related severe multisystem inflammation, began to emerge among pediatric patients. This condition was initially misdiagnosed as Kawasaki disease (KD) and thus was called Kawasaki-like syndrome for its presenting clinical manifestations; only later was it named multisystem inflammatory syndrome in children (MIS-C) [1,2]. This syndrome occurs about four to six weeks after SARS-CoV-2 primary infection [3].

MIS-C is a severe hyperinflammatory disease that manifests in children and adolescents, involving multiple organs and leading to potentially severe, albeit nonspecific, symptoms. It is characterized by the presence of fever, accompanied by gastrointestinal, hematologic, renal, neurologic, and respiratory disorders. Additionally, mucocutaneous manifestations are common, and cardiovascular findings are observed frequently, often culminating in shock. The clinical severity of MIS-C is determined by the number of affected organs and the necessity for intervention. Mild MIS-C refers to cases with minimal organ damage where inotropic or respiratory support is not required. Moderate MIS-C occurs when there is mild or isolated organ injury. Severe MIS-C is diagnosed when moderate to severe organ damage occurs, such as cardiac ventricular dysfunction and shock, necessitating intensive care admission for optimal patient management [4].

The long-term effects of MIS-C are not fully known yet. Initial concerns were mainly focused on cardiovascular damage, particularly coronary involvement, in light of the similarities with KD, and on myocardial injury because of ventricular dysfunction and similarities with viral myocarditis. However, the outcome is mostly favorable. Coronary artery aneurisms are reported to decrease by 79.1% one month after diagnosis and 100% after three months [5]. Myocardial injury rapidly recovers the vast majority of times, particularly systolic function, while mild diastolic dysfunction persists a little longer. Minor abnormalities may be still seen after 1 year in cardiac MRI in children without clinical symptoms [6]. Similarly, the extracardiac effects, such as thromboembolic complications requiring prolonged therapy, are still unknown [7]. Long COVID in children is also an entity that still needs to be fully understood, but so far it seems not to impact MIS-C development.

MIS-C does not directly result from viral infection but rather arises from an abnormal immune response triggered by SARS-CoV-2 antigens, including the spike protein. This immune response can lead to an overactivation of innate immunity, believed to be mediated by the inflammasome. Consequently, a cytokine storm occurs, primarily driven by the overproduction of interleukin-1 (IL-1), tumor necrosis factor-β (TNF-β), and interferon-γ (IFN-γ). Additionally, activation of humoral immunity characterized by a type III hypersensitivity response is observed [8,9]. These immunological processes predominantly affect genetically predisposed individuals, with associations found between MIS-C and specific HLA variants, as well as dysfunction in regulatory genes involved in apoptotic and inflammatory mechanisms [10,11]. 

It is worth noting that these immunological mechanisms may also play a role in other atypical late-onset complications associated with coronavirus disease 2019 (COVID-19), such as chilblains, which have been more frequently described during the initial and subsequent waves of the pandemic [12].

**Figure 1:**
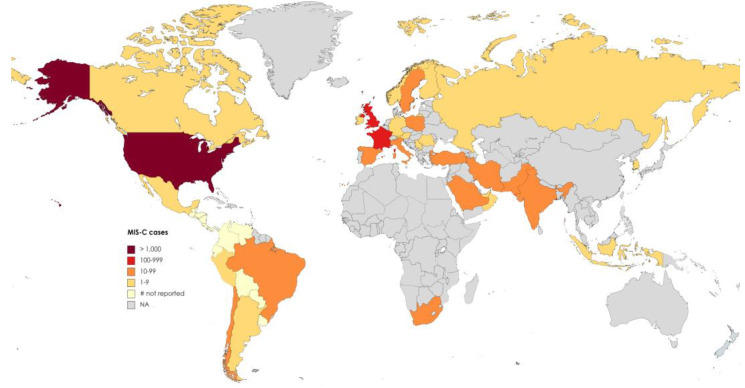
Map of MIS-C cases, by country, as reported in published studies updated up to April 2021. Countries that have reported cases but have not disclosed the number of cases are denoted as “# not reported.” [13].

The incidence rates of MIS-C exhibit variation across different geographic regions. Higher numbers of cases have been reported in Europe, the Americas, Africa, South Asia, and the Middle East, while there is a scarcity of reported cases in East Asian countries. It is likely that environmental factors and social determinants of health disparities, in addition to genetic background, contribute to this geographic distribution of MIS-C incidence [13] (Figure 1).

Based on our clinical experience and the changing patterns of MIS-C, we conducted an analysis of the incidence and severity trends in MIS-C over the months since the beginning of the pandemic, comparing data from various continents. We also aimed to investigate potential immunological factors that could contribute to these changes, considering factors such as vaccination for SARS-CoV-2, the emergence of virus variants, alterations in human immunological responses, and the changes in therapeutic approach. 

To gather relevant information, we conducted a search using major scientific article search services, specifically focusing on English-language articles that evaluated the trend in MIS-C cases over the past few years. Our search employed keywords such as MIS-C, SARS-CoV-2, and Omicron. Furthermore, we reviewed key articles that examined the physiopathological mechanisms underlying MIS-C and SARS-CoV-2 infection to formulate hypotheses regarding how the aforementioned factors may have influenced the behavior of MIS-C in recent months.

## 2. Are We Facing a Global Reduction in the Incidence of MIS-C?

In the past three years, a total of 46 patients who met the diagnostic criteria for MIS-C according to the Centers for Disease Control and Prevention (CDC) were admitted to the pediatric emergency unit of Sant’Orsola Hospital, IRCCS Azienda Ospedaliero-Universitaria in Bologna. This hospital, along with the Pediatric Unit of Ospedale Maggiore, serves a population of approximately 45,000 children between the ages of 0 and 14. Additionally, some patients were evaluated for cardiological consultations while being hospitalized in other regional hospitals. Among the patients admitted to our pediatric unit, 31 cases of MIS-C were identified between April 2020 and July 2021. During this period, SARS-CoV-2 infection could be attributed to pre-Delta/ancestral lineage variants in over 50% of cases, based on data from the Italian Istituto Superiore di Sanità (ISS). From August 2021 to January 2022, only 11 cases of SARS-CoV-2-related MIS-C were reported. During this period, the Delta variant was prevalent in over 50% of cases. After January 2022, four cases of MIS-C were diagnosed. However, since June 2022, no new cases of MIS-C have been detected, coinciding with the last MIS-C patient being identified. During this time, the prevailing variant was the Omicron variant. 

In our clinical experience, we have observed a decrease in MIS-C cases since February 2022, coinciding with the spread of the Omicron variant. The last diagnosis of MIS-C was made in June 2022. Similar findings were reported by Bellini et al. in their study conducted at Gaslini Pediatric Hospital in Genoa, Italy, which is located a few hundred kilometers away from our center. According to Bellini et al., there was a notable reduction in the incidence of MIS-C cases diagnosed at the pediatric emergency department of Gaslini Pediatric Hospital during the peak of the Omicron wave, spanning from December 2021 to March 2022. The incidence rate decreased from 5,080 cases of MIS-C per 100,000 patients with SARS-CoV-2 infection between April 2020 and November 2021 to 1439 cases of MIS-C per 100,000 patients between December 2021 and March 2022. It is important to note that Bellini et al. did not report the patients' median age, vaccination status, or comorbidities in their paper [14].

In the United States of America (USA), the CDC constantly updates and shares MIS-C data since 19 February 2020 (“https://covid.cdc.gov/covid-data-tracker/#mis-national-surveillance”) [15]. Their findings document a decrease in the disease without reports since 28 September 2022; up until 16 October 2022, 9072 cases of MIS-C were diagnosed in the USA and 5558/9072 cases were diagnosed between April 2020 and July 2021, corresponding to 16 MIS-C out of 100.000 subjects infected (i.e., 63.4% of all diagnosed MIS-C in the USA). Between August 2021 and January 2022, there were 7 cases per 100,000 infected (i.e., 30% of all MIS-C), and between February 2022 and October 2022, this number was 3 out of 100,000 (i.e., 8.6% of MIS-C).

In Australia, a total of 107 cases of MIS-C were recorded from May 2020 to April 2022. Of these cases, 5% were associated with the pre-Delta wave (from March 2020 to May 2021), 28% were linked to the Delta wave, and 67% were associated with the Omicron wave. The MIS-C rate decreased from 13 cases per 10,000 during the pre-Delta wave to 5 cases per 10,000 during the Delta wave, further decreasing to 0.8 cases per 10,000 during the Omicron wave [16].

Similar observations have been reported in Europe. In Denmark, Holm et al. found that during the first phase of the pandemic, which was predominantly characterized by pre-Delta variants, the rate of MIS-C was 25 cases per 100,000 individuals. During the Delta wave, the rate increased to 28 cases per 100,000, with the majority of cases (51 out of 52) occurring among unvaccinated people. However, during the Omicron wave, the rate significantly dropped to 2 cases per 100,000, predominantly among unvaccinated subjects. It is worth noting that no cases of MIS-C were diagnosed in patients with SARS-CoV-2 reinfections by Delta and Omicron variants [17].

Data on the association MIS-C and variants are also reported in Germany [18]. The study specifically focuses on MIS-C cases that were confirmed by polymerase chain reaction (PCR) to be attributable to SARS-CoV-2 infection. Between March and August 2021, a total of 143 cases of MIS-C were diagnosed among 231,114 infected patients, resulting in a rate of 62 cases per 100,000 individuals. During the Delta wave, which occurred between August 2021 and January 2022, 291 cases of MIS-C were identified among 1,732,623 infected individuals, corresponding to a rate of 17 cases per 100,000 individuals. From January to April 2022, during the Omicron wave, 97 cases of MIS-C were reported among 3,260,446 infected individuals, resulting in a rate of 3 cases per 100,000 individuals.

In Poland, Ptak et al. evaluated the incidence, the clinical presentation, and the management of all MIS-C patients admitted to the department of pediatrics at the University Children’s Hospital of Cracow between 1 November 2020 and 30 June 2022. When original/Alpha variants were dominant (November 2020–July 2021), 74 (68.5%) out of 108 patients with a suspect of MIS-C were hospitalized, while when the Delta variant was dominant (October 2021–June 2022), 34 patients (31.5%) were hospitalized. Only 3/108 MIS-C patients were hospitalized during the Omicron-variant-domination period. Thus, a decreased incidence of MIS-C was observed despite an increase in the infection rate in Poland during the variants, from original Alpha to Omicron [19].

Levy et al. documented the same trend in Israel. During the Omicron wave, the authors found an incidence of 3.8/100.000, which was indeed far lower than during the previous waves (54.5/100.000 during the Alpha wave; 49.2/100,000 during the Delta wave) [20].

In England, Cohen et al. conducted a study that showed a decrease in MIS-C cases during different waves of the SARS-CoV-2 virus. They found that MIS-C cases were 56% lower during the pre-vaccine Delta period compared to the Alpha period. Furthermore, during the post-vaccine Delta period, MIS-C cases decreased by 66%, and during the Omicron period, they were 95% lower compared to the Alpha variant wave. The incidence rates were reported as follows: 231 cases per 100,000 during the Alpha wave; 102 cases per 100,000 during the pre-vaccine Delta wave; 79 cases per 100,000 during the post-vaccine Delta wave; and 12 cases per 100,000 during the Omicron wave [21]. 

A multicenter international study conducted by Buonsenso et al. also observed a significant global decrease in the ratio between MIS-C cases and SARS-CoV-2 infections during the pre-Delta and Delta waves in various countries worldwide. The authors noted that there were fewer MIS-C diagnoses in countries with higher vaccination coverage against SARS-CoV-2. However, a similar reduction in MIS-C cases was also observed in children who were not yet eligible for vaccination. It is worth mentioning that the majority of MIS-C cases were identified among non-vaccinated children [22].

Notably, the Omicron wave was associated with increased infectivity and transmissibility [23]. Despite this, the rate of MIS-C significantly reduced during the spread of the Omicron variant. Table 1 compares the incidence of MIS-C cases between waves of SARS-CoV-2 in the different studies mentioned above.

## 3. Are We Assisting in the Reduction in the Severity of MIS-C?

**Figure 2:**
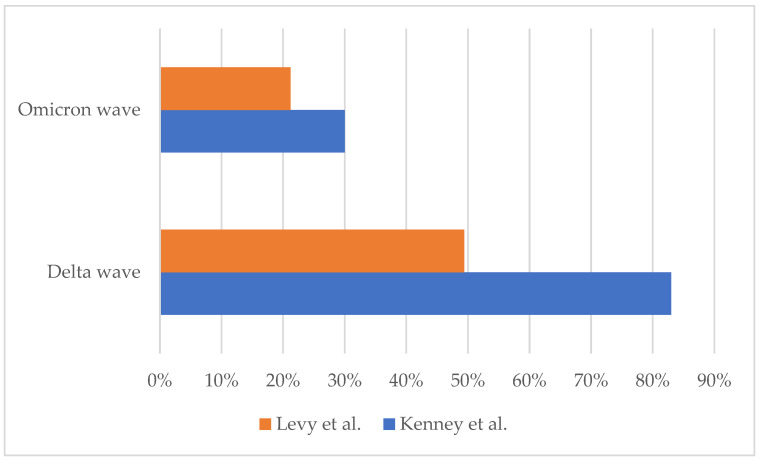
Proportion of patients admitted to pediatric intensive care unit (PICU) out of the total number of MIS-C hospitalizations during the Delta and Omicron waves [20,24].

The severity of the disease follows a similar trend to the incidence of the variant over time. Omicron-related MIS-C cases have milder symptoms and organ damage than previous variants, even though the variant itself is more transmissible. An Israeli study found that MIS-C cases during the Omicron wave were characterized by less severe cardiac damage, shorter hospital stays, and a lower need for intensive care than MIS-C cases during previous waves. The study identified several possible contributing factors, including the Omicron variant, previous infection or vaccination against SARS-CoV-2, and improvements in treatments [20] (Figure 2).

Kenney et al. in early 2023 showed a decrease in the severity of MIS-C during the spread of Omicron compared to the Delta circulation: they reported a significant reduction in shock, duration of hospitalization, and need for vasoactive drugs and intensive care unit (ICU) admission [24] (Figure 2). 

**Figure 3:**
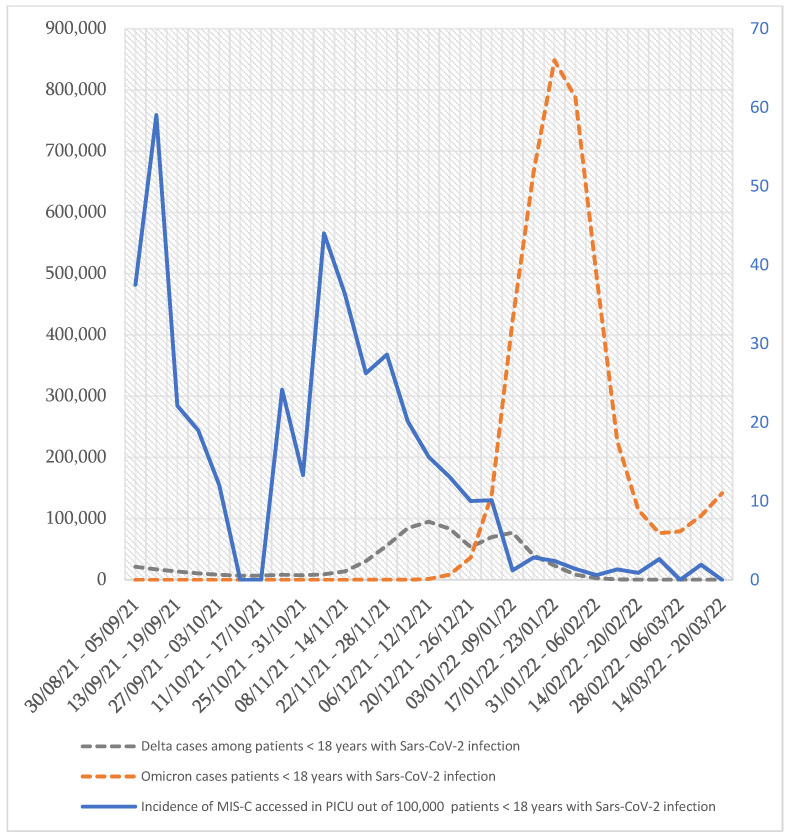
Incidence of MIS-C patients admitted to PICU in relation to Delta and Omicron cases in pediatric positive tested subjects [25]. The values on the *y* axis on the left indicate the number of SARS-CoV-2 cases < 18 years and represent the units of the dashed lines. The values on the *y* axis on the right refer to the incidence of MIS-C cases admitted to PICU and represent the units of the continuous line.

Recher et al., evaluating the risk of admission to the ICU for SARS-CoV-2 Delta and Omicron infections, concluded that the incidence of acute COVID-19 and MIS-C patients admitted to the pediatric ICU were significantly higher with the Delta variant than with the Omicron variant [25] (Figure 3).

Ptak et al. showed that hospitalizations were about two-thirds lower during the Omicron wave compared to the Delta wave. Despite the sample size of patients during Omicron being limited, both groups’ clinical course and severity appeared similar in contrast with the previous observation. These findings are similar to those published by Holm et al., in which no differences were found between the clinical characteristics of MIS-C during the Omicron wave and pre-Omicron wave in Denmark [19].

Interestingly, Abraham et al. in a study recruiting 129 unvaccinated children diagnosed with MIS-C from May 2020 to March 2022 showed that clinical manifestations, laboratory characteristics, and disease evolution did not change during the spread of the new variants in comparison with the ancestral virus, underlining that extreme inflammation remains a virus-related severe complication [26].

Zambrano et al. reported a strong association MIS-C and vaccination; the authors showed a significant decreased in the likelihood of hospitalization for MIS-C in the USA in children aged 12-18 years who had received two doses of 13 BNT162b2 COVID-19 vaccine, mostly during the Delta variant wave, and in children 5-18 either during the Delta or Omicron variants periods compared to unvaccinated children [27].

The debate over the severity of MIS-C cases during the Omicron wave is still ongoing. The available evidence seems to suggest a certain contribution of the vaccine to reducing the severity of MIS-C over time. 

## 4. What Is Contributing to the Change in MIS-C Features over Time?

Studies published so far agree that the incidence rate of MIS-C has significantly decreased worldwide, in line with the spread of the Omicron variant. This observation has been confirmed in very different geographical areas [16,18,19,20]. However, the actual factors contributing to this phenomenon remain unclear. In addition, conflicting data have been published on the severity of the disease during different predominant virus circulations, from the ancestral virus to the Delta variant and then to the Omicron variant [24]. In the following paragraphs, we assess the aforementioned potential factors, starting with the hypothesis on MIS-C pathogenesis.

### 4.1. Elements of Pathogenesis of MIS-C

#### 4.1.1. From SARS-CoV-2 Infection to Persistent Antigenemia

The pathogenesis of MIS-C is still under debate, but like severe COVID-19, MIS-C seems to be driven by immune dysregulation. As previously shown, severe COVID-19 results from a lack of type I and III interferon (IFN) action. In people under the age of 60, severe COVID-19 is observed in those affected by IFN-related inborn errors of immunity. In older people, the proportion of subjects with anti-IFN-I autoantibodies increases, mimicking a lack of IFN action [28].

In MIS-C, the cytokine storm is thought to be the main trigger of clinical manifestations, and that appears to be related to the activity of IFN-γ [13,29]. The initiator of the cascade in MIS-C seems to be the high level of circulating spike protein in the blood of susceptible subjects. This persistent antigenemia, in the context of the negativity of SARS-CoV-2 viremia detected by polymerase chain reaction (PCR), has already been supposed to be the key determinant of the abnormal immune response leading to MIS-C [29]. IFN-γ typically induces presentation of the antigen in various cell types, including monocytes, dendritic cells, and B cells. However, studies have shown that patients with MIS-C have an impaired antigen-presentation ability [30]. This is likely due to the fact that younger patients encounter viruses for the first time, and they display an immune response based on the innate immune system through the cytosolic and membrane-bound pattern-recognition receptors, leading to enhanced IFN signaling. This absence of pre-existing memory cells favors the branch of innate immunity [31]. During early infancy, children constantly face immunological threats with the help of innate immune response. As a consequence, phagocyte and antigen-presenting cells are rapidly activated, and prompt viral clearance is possible [32]. This also may partially explain the lower rate of severe respiratory forms of COVID-19 in children [33]. In older ages, adaptive cell response gradually takes over innate immunity. Type I IFN-based immune response mainly retains an important role as a driver of adaptive immunity, control of local infection, and debris clearance.

Beyond the first years of life, the immune response matures over time to a more adult type. This may represent a window of opportunity for the development of MIS-C [31]. On the one hand, innate immunity, consisting mainly of the patrolling monocytic-macrophage system and natural killer (NK) cells activated by IFN I signaling, is insufficient for viral clearance. On the other hand, adaptive immunity may not be fully efficient. These two aspects may lead to an altered pathway of antibody production and affinity maturation, with the result of persisting antigenemia.

#### 4.1.2. From Antigenemia to Altered Antibody Production and Inflammation

Antigenemia is not sufficient to initiate MIS-C, but adaptive immunity is also thought to play a role. This is supported by observations of patients with inborn errors of immunity who have experienced mild COVID-19. These patients have adaptive immune deficiencies, but their disease course was comparable with or even less symptomatic than the general population. This suggests that certain components of adaptive immunity are not essential for controlling SARS-CoV-2 infection, and that adaptive immune deficiencies may even contribute to a milder course by reducing immune-mediated sequelae [34,35,36]. MIS-C occurs most commonly in pre-adolescent patients, and there is a latency period between SARS-CoV-2 infection and the onset of MIS-C. This suggests that MIS-C may be related not only to antigenemia, but also to a unique pathway of antibody production [37]. 

Children older than one year of age who are infected with SARS-CoV-2 or who have been exposed to the virus may produce a proportion of non-neutralizing antibodies to the SARS-CoV-2 surface protein, in addition to neutralizing antibodies to the N-terminal domain and receptor-binding domain (RBD). This may paradoxically worsen the viral infection through a process called antibody-dependent enhancement (ADE) [38,39].

ADE is a mechanism in which low-titer, non-neutralizing antibodies (either natural, cross-reactive, or low-affinity) can bind to a virus and facilitate its entry into cells of the reticuloendothelial system via the Fc receptor. In the case of SARS-CoV-2, this can lead to the internalization of a virus that has not yet been activated by cleavage of the spike protein. The presence of viral components in the cytosol can then trigger an inflammatory response and the production of pro-inflammatory cytokines [18]. This process has already been observed as a cause of increased disease severity, such as dengue and Zika infections, sustained by viruses that show similarities in the structure of their non-neutralizable epitopes, determining cross-reaction-driven ADE [40]. Even though ADE has not been associated with COVID-19 pathogenesis, an imbalance resulting in poorer neutralizing antibody formation in children (both in quality and quantity) may also delay viral clearance and perpetrate SARS-CoV-2 antigenemia [38]. The progression from SARS-CoV-2 infection to MIS-C is not yet fully understood, but the observation that MIS-C typically occurs four weeks after SARS-CoV-2 exposure suggests that there may be a minimum threshold of antibody–antigen interactions that must be exceeded before inflammation manifests clinically. Antibodies peak at day 22 for IgA and between weeks 2 and 5 for IgG after infection [41]. MIS-C only develops in patients who are able to produce a significant antibody response but with an imbalance towards non-neutralizing antibodies. This imbalance could favor ADE, as evidenced by the finding that SARS-CoV-2 opsonized with non-neutralizing antibodies can induce inflammation when internalized by macrophages [39].

Severe and prolonged infections are known to produce higher titers of neutralizing antibodies than milder cases. This is likely due to the prolonged antigenic stimulation that occurs during these infections. The magnitude of antibody production seems to reflect the severity of the infection. In children, the infection is often limited to the upper airway due to the predominant stimulation of an innate interferon-based immune response. This means that seroconversion may be lower and the disease induced by SARS-CoV-2 may be milder [42]. Older children may be able to produce antibodies above the threshold needed for MIS-C initiation [31].

The role of cellular immunity has to be underlined. When SARS-CoV-2 escapes innate response, either by lack of IFN I action or non-canonical internalization, the virus may replicate in the entry site and fail to prime the adaptive response. This delays the production of highly affine and neutralizing antibodies guided by T lymphocytes. Specific CD4+ T cells have indeed been previously associated with the resolution of SARS-CoV-2 infections [43] and with the rise in neutralizing antibody titers. Conversely, the absolute lymphocyte count has been found to be lower in patients with severe COVID-19 and MIS-C [44], even compared to healthy controls [37].

CD4+ T subpopulations and their induction and maturation during acute infections play a role in the path to immunopathology. Adult patients hospitalized for severe COVID-19 showed a predominant Th1 signature with substantial production of IFN-γ [45]. Even in children, IFN-γ has been found at higher levels in MIS-C than in patients with COVID-19 and healthy patients. Observations indicate that Th1 response seems predominant over Th2 signature in acute phases of severe COVID and MIS-C. The former is linked to inflammation, further Th1 expansion, and inhibition of Th2 differentiation; the latter is associated with the path of antibody production, Ig class switch, and immune-response modulation. The imbalance between Th1 and Th2 and the absence of progression to an adaptive immune response may also force the host to rely only on innate immunity to control infection, causing immune-mediated tissue damage.

CD8+ cells are less expressed than CD4+ T cells during the acute phase and convalescence of MIS-C [37,46], and in adults, the majority of patients display a CD8+ T cells able to produce IFN-γ and granzyme, together worsening the immune damage. At this point, another path of increased inflammation is worth mentioning. Chronic infection would bring an exhaustion of CD8+ T cells [29], a phenomenon in which inhibitory signals prevail to minimize immunopathology, which has been already highlighted in COVID-19 [47]. Persistent antigenemia, as encountered in MIS-C patients, without productive SARS-CoV-2 infection, may alter the induction of exhausted CD8+T cells and bring them to cause immunopathology.

### 4.2. The Role of the Immune System in the Change in MIS-C Epidemiology

The spread of variants with reduced morbidity and mortality suggests that the inflammation threshold needed to initiate MIS-C is now more difficult to reach. This may be due to a rapid adaptive immune response, both cellular and humoral, that is favored by cross-reactivity between the epitopes of different variants, which speeds up the production of neutralizing antibodies.

**Figure 4:**
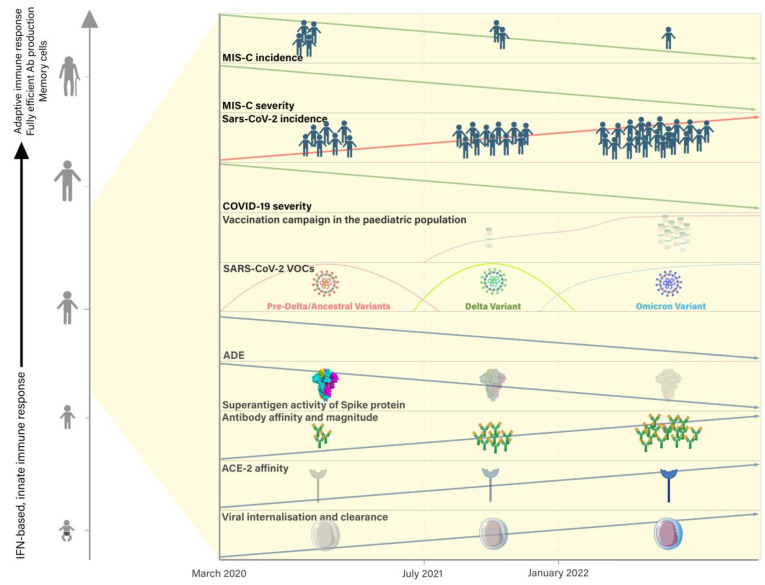
Trend in SARS-CoV-2 and MIS-C incidence and severity during the SARS-CoV-2 pandemic waves (pre-Delta/ancestral wave, Delta wave, and Omicron wave) and spread of COVID vaccination among pediatric population. Change in interaction between the virus and the immunological system from the beginning of pandemic to today. VOCs, variants of concern; ADE, antibody-dependent enhancement; ACE-2, angiotensin-converting Enzyme-2.

SARS-CoV-2 variants are closely related and many of their structural components have similar epitopes [48]. Antibodies to these epitopes may have matured over time, leading to a higher concentration of neutralizing antibodies on the surface of SARS-CoV-2 [38]. This would have increased the ability of antibodies to neutralize viral particles and be phagocytosed by patrolling macrophages. In conclusion, the affinity maturation of antibodies may have quickly reinforced immunity throughout the pandemic, and ADE based on non-neutralizing, poorly affine antibodies may have gradually waned (Figure 4).

The affinity of antibodies, the magnitude of detectable titers, and their duration may all have influenced the pathway to MIS-C. It is also worth noting that the gradual easing of social distancing has increased the spread of the virus. As expected, the risk of MIS-C initially increased in line with the epidemiology of the virus, but then declined, as shown in recent studies [21,27]. Evidence suggests that immunity to other coronaviruses wanes within 3 years of natural infection [49]. One study found that only 13% of SARS-CoV-2 patients lost their IgG titers after 10 months, meaning that most patients still have detectable titers at the start of the following winter [50]. In children, titers after natural infection have been detectable for up to 9 months [42]. The presence of pre-existing memory in B cells that produce antibodies with sufficient affinity suggests that children may have a certain degree of tolerance to MIS-C, possibly due to a significant concentration of preformed antibodies.

Finally, it is worth considering SARS-CoV-2-induced specific T cells. CD4+T cells show cross-reactivity among another beta coronavirus, and even younger children show pre-existing T cells that are reactive to SARS-CoV-2 due to endemic circulation of coronavirus in infancy [45,51,52]. During infancy, CD4+ T cells are often induced in response to cross-reactive viral epitopes. This helps to reduce the severity of respiratory infections, including coronaviruses, and promotes the development of a prompt adaptive immune response. Although the role of CD4+ T cells in SARS-CoV-2 infection is not fully understood, they appear to boost the host’s response to the virus, helping to clear it from the body [53]. 

Memory B and T cells are found in the blood of fully recovered patients with a previous COVID-19 infection, and the latter showed similar a expression profile to the acute phase [54]. Children, after multiple exposures to SARS-CoV-2, are able to induce a population of immune cells ready to initiate mature humoral and cellular response. These may explain not only the drop in the incidence of MIS-C, but also the reason MIS-C has not been shown to recur with reinfection with SARS-CoV-2 [55,56].

### 4.3. The Role of Variants of SARS-CoV-2 (VOCs) in the Change in MIS-C Epidemiology

VOCs are distinguished by changes in the spike surface protein. The Omicron variant has the most mutations in this protein compared to the original virus (both Alpha and other pre-Delta variants) [36]. These mutations are concentrated in a region that is structurally similar to a region that was previously identified as a “superantigen”. Superantigens can interact with polyclonal T cells, which are T cells that express a wide variety of T cell receptors. This interaction can promote the expansion of these T cells and the production of cytokines [57]. Although a recent study by Amormino et al. found that the SARS-CoV-2 spike protein does not have intrinsic superantigen-like inflammatory activity, it is reasonable to assume that mutations in the spike protein, as well as mutations in other SARS-CoV-2 proteins involved in the pathogenesis of MIS-C, could gradually lead to a loss of pro-inflammatory activity in these proteins [58,59].

In fact, even considering that each gene within the SARS-CoV-2 genome exhibits a distinct mutation rate, the mean rate of mutation of the SARS-CoV-2 genome is relatively slower than most RNA viruses, estimated to be around 8 × 10^−4^ or potentially as high as 1.37 × 10^−3^ substitutions per site per year. It is worth noting that each gene within the SARS-CoV-2 genome exhibits a distinct mutation rate. Notably, the spike region demonstrates the highest mutation rate, with approximately 1.34 × 10^−2^ substitutions per site per year [60]. Despite that, Omicron and other non-Alpha variants show a percentage of similarity between their spike protein greater than 90%, even though some difference has to be highlighted. Omicron, compared to Delta, has a higher number of charged amino acids that allow the formation of a greater number of salt bridges and improve interaction with ACE-2 [61]. In addition, the Omicron spike showed the greatest number of strong binders of T cells compared to Delta and other pre-Delta variants. This means that Omicron retains the predicted highest ability to generate antibodies by MHC II presentation and trigger adaptive immunity [62]. Moreover, except from the Alpha variant, the difference in the tertiary structure of spike between Omicron and Delta has shown to be greatest among other pre-Delta variants, and that may change host–pathogen interactions significantly. These latter variants showed greater affinity with angiotensin-converting enzyme 2 (ACE-2) and accumulated mutations in the neutralizable N-terminal domain of spike protein compared to the original and Alpha variant. This first aspect has potential implications for host–pathogen interactions. Thanks to a high-affinity interaction between the receptor-binding domain (RBD) of spike protein and ACE-2, and due to a greater expression of ACE-2 [63] in younger individuals, a rapid internalization and intracellular cleavage of SARS-CoV-2 spike protein may be warranted Recent studies have shown that specific charged amino acid substitutions in the RBD, S1, and S2 domains of new SARS-CoV-2 variants may have contributed to this increased affinity. One possible explanation for this is that positively charged amino acids may have facilitated a stronger and easier interaction between the virus and the negatively charged human cell surface [64,65]. Additionally, the expression of ACE-2 in humans is induced by interferons (IFNs), especially in young children [66]. Given the physiological predominance of the innate immune system over the adaptive immune system, IFNs are frequently induced by novel viral antigens. This results in intracellular infection and subsequent rapid viral clearance from the entry site, preventing the virus and its debris from remaining in the extracellular space and interacting with low-affinity non-neutralizing antibodies.

The N-terminal domain (NTD) of the spike protein accounts for about 35% of the antibody response to SARS-CoV-2. However, in the case of Omicron, only about one-third of these antibodies are neutralizing [62]. SARS-CoV-2 is a neutralization-sensitive virus. Although variants have accumulated mutations in critical epitopes from the original virus, antibody responses in humans can only control infection if they are produced at a sufficient concentration. It is possible that mutations of epitopes in the NTD have become the target of neutralization. This has been explored with antibodies that target different epitopes, few of which have been shown to be preserved among VOCs [67,68]. Clearly, antibodies that target the RBD of spike are able to prevent the interaction though ACE-2 on the cell wall, reaching neutralization, but other sites of spike have gained interest. In particular, different antibodies are able to recognize similar spatial (tertiary) epitopes, in a region of spike identified as a supersite, that prevent interaction and invasion of the cell [62]. Few other antibodies have shown the interesting ability to bind specifically to different epitopes than the supersite. In particular, COV2-3434 is able to bind to NTD in a region of interaction that is needed for spike trimers’ formation, inhibiting transmission from cell to cell. Therefore, sites of NTD may become a potential target for vaccine development, as more preserved epitopes are discovered that allow neutralization.

### 4.4. The Role of Vaccination in the Change in MIS-C Epidemiology

From 2021 to 2023, among the pediatric population in the United States, 72.9% of children between 5 and 12 years old and 77% of adolescents aged 12 to 18 years have received at least two doses of vaccine against SARS-CoV-2 [69]. 

A decrease in the incidence of MIS-C during the Delta and the Omicron waves and a protective effect due to vaccination has been shown [16,17,27]. In the fully vaccinated pediatric population, disease incidence is estimated to be 0.3 MIS-C cases per million children [70]. Fully vaccinated patients with MIS-C require less respiratory or cardiovascular support, as opposed to 39% of unvaccinated MIS-C patients. Moreover, the rate of fully vaccinated patients admitted to the intensive care unit was 20% vs 69% of unvaccinated patients [27].

As of today, it remains a very rare complication in this group, and describing a clinical profile remains complicated. However, what we can refer to are studies comparing MIS-C during the Omicron period, during which the vaccinated pediatric population was higher, with that of the Delta variant. As reported above, what emerges is a milder clinical picture with a faster course.

mRNA-based vaccines are the main type of SARS-CoV-2 vaccine used in children in Europe and the United States. These vaccines encode information for the synthesis of the SARS-CoV-2 spike protein, which is then recognized by the immune system. The correlate of vaccine efficacy and protection is the presence of neutralizing antibodies in the blood. Both adults and children can produce a broad range of neutralizing antibodies against the spike protein [71,72,73]. In a low-incidence setting of infection, children (mean age 8.3) have higher antibody levels and their immunity seems to wane more slowly than adults. These observations suggest that children are generally able to mount a strong immune response to the virus and its components [21].

Vaccination elicits an immune response that targets the spike protein of SARS-CoV-2. This response leads to neutralization of the virus, decreased pathogenicity, and increased antibody concentration with age [74]. Natural infection, on the other hand, can lead to a broader range of antibodies, including anti-nucleocapsid and non-neutralizing antibodies. These antibodies may be the basis for antibody-dependent enhancement (ADE) and potentially multisystem inflammatory syndrome in children (MIS-C). Vaccination, on the other hand, is more likely to lead to a strong and neutralizing antibody response, which is associated with a lower risk of ADE.

The massive vaccination campaign and the continuous global spread of the infection have led to an increase in the number of people with SARS-CoV-2-induced cell-mediated adaptive response. This specific immune memory could help to reduce the severity of COVID-19 by switching from an innate immune response to a more tailored response to the virus.

After this consideration, it may be debated that in naive and unvaccinated individuals, such as unvaccinated infants facing a SARS-CoV-2 infection for the first time, incidence of MIS-C should not show any difference irrespective of the infectious variants. Accordingly, MIS-C would greatly change its epidemiology, with infants and young children prevailing in number over pre-adolescents and being proportionally more prone to severe forms of MIS-C than older children. In fact, a change in epidemiology has been already observed in a recent study, with children displaying MIS-C at a younger age than with previous variants. However, the clinical characteristics of MIS-C have been shown to resemble Kawasaki in younger patients, likely reflecting a different stereotyped response to SARS-CoV-2 antigens in this age group [75]. This switch may reflect a potential effect of vaccination in this population.

Given that vaccine-induced antibodies lose efficacy against different VOCs in adults with symptomatic COVID-19, it is worth wondering why we do not see the same loss of efficacy in preventing MIS-C [56]. In our experience and to our knowledge, the incidence of MIS-C in infants has always been extremely low throughout the circulation of different variants. Additionally, previous reports have not shown a difference in the median age of children with MIS-C during different pandemic waves [12,13,17]. 

Eventually, both vaccines and natural infection induce T cells that recognize VOCs by cross-reacting with intracellular epitopes [55]. It is worth wondering why antibodies show a loss of efficacy against different VOCs in adults with symptomatic COVID-19 but are still able to prevent MIS-C [56]. Furthermore, it should be noted that FDA approved bivalent mRNA vaccines in infants only in December 2022, when the tendency to a lower incidence and severity of MIS-C had already been observed. As mentioned earlier, antibody-dependent enhancement (ADE) is induced by antibodies that are poorly neutralizing. This has also been shown to be a complication of other vaccines, such as the dengue vaccine. In these cases, the morbidity of dengue was enhanced in patients who had received the vaccine the previous year. The antigenic variability of the S protein in the context of new variants of concern (VOCs) may actually enhance immune-mediated pathology when an individual is exposed to a different variant of SARS-CoV-2. This is because subsequent infections from different variants can elicit memory B cells to produce antibodies that initially targeted different epitopes, but now have little neutralizing activity against the current infection. In fact, even though mRNA vaccines are constantly redesigned to match variants of the S protein, the overall antibody response is polyclonal and directed to epitopes that are distant from the receptor-binding domain [76]. Despite the loss of neutralization, the lower disease burden of MIS-C in the era of VOCs suggests that differences in viral components may have a smaller impact on the pathogenesis of MIS-C. Additionally, natural infection that may occur in vaccinated individuals induces not only antibodies that recognize epitopes of the S protein, but also other surface antigens and T cells that bind intracellular epitopes that cross-react among VOCs [77], enhancing antiviral response and clearance.

These considerations support the hypothesis that the magnitude of the overall antibody and cellular response may be more important than the epitopes of the VOCs that may escape neutralization. Therefore, it may be suggested that the change in MIS-C epidemiology has less to do with the intrinsic characteristics of the virus than with other factors, such as the effectiveness of vaccines and natural infection.

Another consideration of the severity of MIS-C is worth mentioning. As reported above, Abraham et al. showed that clinical manifestations, laboratory characteristics, and disease evolution have not changed during the spread of new variants compared to the ancestral virus in unvaccinated children diagnosed with MIS-C from 2020 to 2022 [16]. On the other hand, a study in Israel, where a massive vaccination campaign has drastically decreased the number of naive individuals, noted that MIS-C cases during the Omicron wave were characterized by less severe cardiac damage, shorter hospitalization, and a lower need for intensive care [20]. Other reports are in line with these findings [21,24].

Even though with vaccination patients only produce antibodies against spike, a proportion of them target the NTD rather than the RBD. These attach to epitopes that warrant neutralization and may have contributed to the reduced susceptibility to severe COVID-19 in adults [67]. The possibility of producing such neutralizing antibodies has not been shown with natural infections. This is likely due to differences in the tertiary structure of the natural spike to the spike transcripted from the genetic material of vaccines.

Clarifying whether vaccination with a single component of the original virus, two or more variants, or natural infection all contribute equally to the decline in MIS-C rates would necessitate a change in public health policies. The risk-to-benefit ratio of mRNA vaccines must be re-evaluated if their contribution to MIS-C reduction (in incidence and severity) is equal to that of natural infection, even if there is a slight increase in myocarditis and arrhythmias [78,79].

To date, no studies have been published that compare vaccines and schedules with respect to variants, nor have any studies used MIS-C characteristics as a hard endpoint. For example, a well-designed study by Zambrano et al. demonstrated the efficacy of a two-dose mRNA vaccine schedule over single-dose and unvaccinated individuals, both in terms of MIS-C incidence and severity. However, the study did not include analyses of VOCs or comparisons with other schedules [27,80].

Young children may benefit from continuous exposure to SARS-CoV-2 antigens and immune-response maturation, as well as the vaccination campaign. Other approaches may also be explored. Models based on Brownian motion, which were originally developed to describe the growth of self-reproducing phenomena [81], can be applied to pandemics to describe the cumulative number of COVID-19 cases in a community or fluctuations in the reproduction number over the course of a season [82]. These models exhibit interesting properties when they are subjected to a “reset”, which is an event that halts the ongoing dynamics and restarts the entire process. In this case, such events could be represented by the isolation of a family group or a general lockdown. Studies have shown that these systems are non-ergodic [83], meaning that they will not always be trapped in a small number of configurations. The probability of the model exhibiting extreme values has been shown to be inversely related to the “resetting rate” [82]. Containment measures and isolation may have prevented unexpected variations in the pandemic dynamics during the early stages. However, a similar approach may not always be feasible today. In the event of an emerging MIS-C cluster, increased COVID-19 severity, or a critical shortage of healthcare resources, rapid and frequent containment measures to reset the dynamics of the pandemic would result in fewer variations than a softer approach.

### 4.5. The Role of Therapeutic Novelties in the Change in MIS-C Severity

The first-line therapy for MIS-C traditionally consists of a combination of intravenous immunoglobulins, glucocorticoids, and monoclonal antibodies such as anakinra. Additionally, the use of low-dose aspirin (3–5 mg/kg/day) is recommended in all hospitalized patients with MIS-C, unless contraindicated. This treatment should be continued for at least one month from the diagnosis or until coronary abnormalities and laboratory abnormalities are resolved. In addition to aspirin, the concomitant and prophylactic use of anticoagulants is indicated in presence of general risk factors for thromboembolism or when echocardiographic abnormalities are detected, such as mild to moderate ventricular dysfunction, coronary dilation/aneurysm with a z-score between 2.5 and 10, D-dimer levels of 5 to 10 times the upper limit of normal, thromboelastography in maximum amplitude equal to or greater than 80 mm, or any new significant rhythm abnormalities, like heart block, premature atrial and ventricular contractions, conduction abnormalities, and ST-segment changes [84,85]. 

It appears that the therapy for MIS-C has not undergone significant changes over the years and still relies on the aforementioned pharmacological cornerstones. Thrombotic events complicate the course of patients hospitalized with MIS-C showing greater mortality than patients free from such events [86]. Unfortunately, most studies comparing the effect of anticoagulation therapy do not distinguish severe COVID and MIS-C, but they agree on the potential benefit [87,88]. Therefore, it has not been reported that reduction in severity of MIS-C may be attributable to therapeutic advancements in the field of MIS-C.

## 5. Limitation

When conducting this research, it is necessary to consider possible limitations. First of all, there is significant variability among populations participating, such as selection criteria, the diagnostic protocols, and the type of diagnostic test. Second, uncertainties on MIS-C pathogenesis and conflicting results regarding factors contributing to MIS-C epidemiology have emerged [24]. The lack of available data on MIS-C contribute to leaving the gap still open.

## 6. Conclusions

A combination of factors can be responsible for reduction in MIS-C severity and incidence through time, and more particularly between the Delta and Omicron wave: maturation of an immunological memory to SARS-CoV-2 over time, the implication of mutations of key amino acids of S protein in VOCs, the overall immune response elicited by vaccination over the loss of neutralization of vaccines to VOCs. These all play an important role in this change. However, it is difficult to precisely quantify each factor’s role. First, only a few papers describe MIS-C and the virus variants. Second, the immune state of MIS-C patients is nowadays heterogeneous: it is difficult, indeed, to distinguish the cases of MIS-C in patients with primary infection from those due to SARS-CoV2 reinfection and from those occurring in vaccinated subjects who were subsequently infected. Evidence suggests that each of the factors described may potentially bring a drop of MIS-C incidence and virulence, despite the increase in SARS-CoV-2 cases in the pediatric population, which have drastically raised after the relaxation of social measures. Concerning the risk of severe COVID-19 and MIS-C during the spread of Omicron, rare innate immune defects have been identified as a predisposing factor for MIS-C, enhancing the importance of the early mechanism of viral containment. Notably, there are other common factors that influence the functionality of innate immunity (e.g., epigenetic factors, HLA loci, the association of common variants of crucial genes, autoantibodies against IFN, inborn error of immunity) that may play a peculiar role in severe COVID-19 and MIS-C and in the rest of the population.

## Figures and Tables

**Table 1:** Incidence of MIS-C cases between waves of SARS-CoV-2 in different countries. Incidence varies depending on the sample chosen in different studies. Numbers do not always represent the overall incidence in general population.

	SARS-COV-2 Wave in Relation to the Variant		
	Pre-Delta Wave	Delta Wave	Omicron Wave
**USA** [15]	16/100.000	7/100.000	3/100.000
**Australia** [16]	1.3/100.000	0.5/100.000	0.08/100.000
**Denmark** [17]	25/100.000	28/100.000	2/100.000
**Germany** [18]	62/100.000	17/100.000	3/100.000
**Israel** [20]	54.5/100.000	49.2/100.000	3.8/100.000
**England** [21]	231/100.000	79–102/100.000	12/100.000
